# *BRAF*-mutant high-grade glioma with pleomorphic and pseudopapillary features (HPAP): A PLNTY mimic demonstrating tumor progression during longitudinal follow-up

**DOI:** 10.1093/noajnl/vdag008

**Published:** 2026-01-18

**Authors:** Yutaro Takayama, Mariko Yaegashi, Kaishi Satomi, Takahiro Ishiyama, Masao Shioda, Osamu Yazawa, WeiKai Ye, Hinako Okagawa, Shuna Saito, Kanoko Sasaoka, Miyu Okura, Akihide Koyama, Akito Oshima, Masaki Sonoda, Manabu Natsumeda, Koichi Ichimura, Shoji Yamanaka, Satoshi Fujii, Tetsuya Yamamoto, Kensuke Tateishi

**Affiliations:** Department of Neurosurgery, Yokohama City University, Graduate School of Medicine, Yokohama; Department of Neurosurgery, Yokohama City University, Graduate School of Medicine, Yokohama; Department of Pathology, Kyorin University Faculty of Medicine, Tokyo; Department of Molecular Pathology, Yokohama City University, Graduate School of Medicine, Yokohama; Department of Neurosurgery, Yokohama City University, Graduate School of Medicine, Yokohama; Department of Neurosurgery, Yokohama City University, Graduate School of Medicine, Yokohama; Laboratory of Biopharmaceutical and Regenerative Science, Graduate School of Medical Science, Yokohama City University, Yokohama; Laboratory of Biopharmaceutical and Regenerative Science, Graduate School of Medical Science, Yokohama City University, Yokohama; Laboratory of Biopharmaceutical and Regenerative Science, Graduate School of Medical Science, Yokohama City University, Yokohama; Department of Neurosurgery, Yokohama City University, Graduate School of Medicine, Yokohama; Laboratory of Biopharmaceutical and Regenerative Science, Graduate School of Medical Science, Yokohama City University, Yokohama; Laboratory of Biopharmaceutical and Regenerative Science, Graduate School of Medical Science, Yokohama City University, Yokohama; Department of Legal Medicine, Graduate School of Medical and Dental Science, Niigata University, Niigata; Department of Neurosurgery, Yokohama City University, Graduate School of Medicine, Yokohama; Department of Clinical Cancer Genomics, Yokohama City University, Yokohama; Department of Neurosurgery, Yokohama City University, Graduate School of Medicine, Yokohama; Department of Neurosurgery, Brain Research Institute, Niigata University, Niigata; Department of Brain Tumor Biology, Brain Research Institute, Niigata University, Niigata; Department of Pathology, Kyorin University Faculty of Medicine, Tokyo; Department of Diagnostic Pathology, Yokohama City University Hospital, Yokohama; Department of Molecular Pathology, Yokohama City University, Graduate School of Medicine, Yokohama; Department of Diagnostic Pathology, Yokohama City University Hospital, Yokohama; Department of Neurosurgery, Yokohama City University, Graduate School of Medicine, Yokohama; Department of Neurosurgery, Yokohama City University, Graduate School of Medicine, Yokohama; Laboratory of Biopharmaceutical and Regenerative Science, Graduate School of Medical Science, Yokohama City University, Yokohama

**Keywords:** HPAP, *BRAF*, PLNTY, *TERT*, DNA methylation classifier

## Abstract

**Background:**

High-grade glioma with pleomorphic and pseudopapillary features (HPAP) is a recently recognized glioma subtype defined by DNA methylation profiling. While it exhibits overlapping histological features with various CNS tumors, such as polymorphous low-grade neuroepithelial tumor of the young (PLNTY) and pleomorphic xanthoastrocytoma, its molecular pathogenesis and clinical behavior remain incompletely understood.

**Case Presentation:**

We report a rare case of HPAP with BRAF p.V600E mutation and PLNTY-like histological features that showed rapid tumor progression during long-term follow-up. A 47-year-old woman harbored a lesion that remained asymptomatic and slow-growing for over 20 years, but later exhibited contrast enhancement and rapid expansion. Partial tumor resection was performed with hippocampal preservation based on intraoperative genetic testing and functional considerations. No regrowth of the residual hippocampal lesion was observed at 12 months postoperatively. Histologically, the tumor showed oligodendroglioma-like morphology, strong CD34 immunopositivity, consistent with PLNTY-like features, but indicated a high proliferative index. Molecular analysis revealed co-occurring BRAF p.V600E and TERT promoter (pTERT, c.-124C>T) mutations, with a lower variant allele frequency for the pTERT mutation. This disparity, confirmed by droplet digital PCR, suggests that the BRAF p.V600E mutation was an early, clonal event, whereas the pTERT mutation likely arose later in a subclonal population. DNA methylation profiling classified the tumor as HPAP with high confidence (NCI-Bethesda score: 0.969), and uniform manifold approximation and projection showed clustering within HPAP reference cases.

**Conclusion:**

This case represents a rare example of BRAF p.V600E-mutant HPAP with PLNTY-like features in which a subclonal pTERT mutation likely emerged during tumor evolution, contributing to rapid tumor progression. The combination of a prolonged indolent phase followed by rapid growth, along with the intratumoral genetic heterogeneity observed, provides novel insights into the biological diversity and evolutionary dynamics of HPAP.

Key Points This case demonstrates that BRAF p.V600E–mutant HPAP can closely mimic PLNTY histologically, yet show distinctly more aggressive behavior; DNA methylation profiling (NCI-Bethesda classifier) is essential for accurate classification.

Longitudinal follow-up revealed a prolonged indolent phase followed by rapid progression associated with TERT promoter mutation and chromosomal instability, suggesting stepwise malignant evolution within HPAP.

Patient-derived tumor cells showed sensitivity to BRAF inhibition, supporting the potential role of molecular targeted therapy in selected BRAF-mutant HPAP cases.

High-grade glioma with pleomorphic and pseudopapillary features (HPAP) has been recognized as a novel methylation-defined class of relatively circumscribed gliomas.^1^ Currently, HPAP can only be reliably identified using the NCI-Bethesda CNS tumor classifier (v2) (Laboratory of Pathology, NCI, accessed via Methylscape; https://methylscape.ccr.cancer.gov/, accessed October 5th, 2025), and only 37 cases have been reported across four publications to date.^1-4^ Here, we report a unique case of *BRAF* p. V600E-mutant HPAP that exhibited polymorphous low-grade neuroepithelial tumor of the young (PLNTY)-like features. Longitudinal follow-up revealed aggressive clinical behavior, and molecular analysis identified a *TERT* promoter (*pTERT*) mutation and chromosomal instability in the tumor.

## Case Report

**Figure 1.**
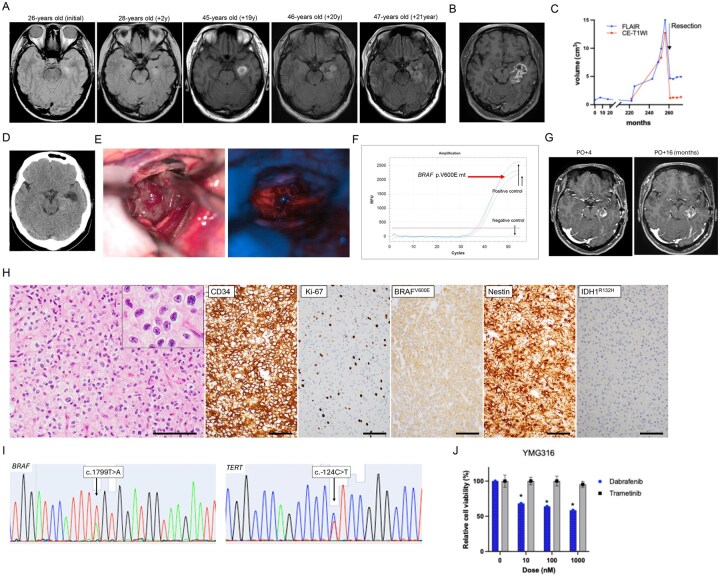
Clinicopathological Features. (A) Axial FLAIR images acquired at the indicated months from initial imaging (displayed from left to right) demonstrate progressive enlargement of the lesion, extending from the mesial temporal structures toward the lateral cortex. Tumor growth accelerated 20 years after initial MRI. (B) Preoperative T1-weighted contrast-enhanced MRI demonstrated that the tumor was primarily located in the mesial temporal structures, including the hippocampus and amygdala, with associated contrast enhancement. (C) Longitudinal changes in tumor volume measured on FLAIR (blue) and contrast-enhanced T1-weighted imaging (CE-T1WI, red). A marked reduction in volume was observed following surgical resection. The residual volume after surgery corresponds to the preserved hippocampus. A focal increase in volume outside the hippocampus was seen before surgery. Postoperative volume increase was not observed. (D) No evident calcification was observed on CT. (E) Intraoperative image: The tumor appeared grayish-white, soft, and highly vascularized. Fluorescence imaging with 5-aminolevulinic acid revealed a strong signal within the tumor tissue. (F) *BRAF* p. V600E mutation was confirmed by real-time PCR, with amplification curves indicating the presence of the mutant allele (red arrow). (G) Postoperative T1-weighted contrast-enhanced MRI (left; post-operative (PO) 4 months (M), right; PO 16M). (H) Histopathological examination shows tumor cells with oligodendroglioma-like morphology on hematoxylin and eosin staining. Immunohistochemistry for the indicated proteins. The Ki-67 labeling index is 20%. Bars, 100μm. (I) Sanger sequencing demonstrates a *BRAF* p. V600E mutation (left) and a *TERT* c.-124C>T mutation (right). (J) In vitro drug sensitivity assay of YMG316 patient-derived tumor cells. Cell viability assays were performed on primary tumor cells after 3 days of treatment with dabrafenib or trametinib at indicated concentrations. DMSO, control. * *P* < 0.05. Data are represented as the mean ± SEM.

A 26-year-old right-handed woman (YMG316) previously underwent stereotactic radiotherapy for a right parietal arteriovenous malformation. During MRI for radiosurgical planning, an incidental abnormal signal was detected in the left hippocampus (Figure 1A). As the lesion was asymptomatic, she was placed under regular imaging surveillance. At age 45, the patient experienced an incidental hemorrhage in the left medial temporal lobe. Serial imaging demonstrated that the hemorrhage remained stable without expansion. At age 46, she developed transient aphasia, and MRI revealed enlargement of the previously identified abnormal signal, now extending into the temporal stem. By age 47, further progression was noted, although the original hippocampal component remained unchanged. Gadolinium-enhanced MRI demonstrated irregular contrast enhancement (Figure 1B;  Figure S1A), and volumetric analysis showed a marked acceleration in tumor growth compared with the preceding indolent phase (Figure 1C). These findings suggested partial malignant transformation of a long-standing low-grade lesion. Remarkably, the patient remained seizure-free throughout the 21-year observation period.

Preoperative imaging demonstrated tumor infiltration extending from the hippocampus into the adjacent temporal white matter. CT imaging revealed no calcification (Figure 1D). Functional assessments showed left hemisphere language dominance on Wada testing, and the Wechsler Memory Scale-Revised indicated mildly reduced verbal memory relative to visual memory. Given the patient’s strong desire to preserve memory function, particularly verbal memory, we planned hippocampal preservation contingent upon intraoperative findings. Craniotomy and tumor resection were subsequently performed (Figure 1E; Figure S1B). Photodynamic diagnosis using 5-aminolevulinic acid was strongly positive (Figure 1E). Intraoperative frozen sections suggested a low-grade neoplasm. Using our original intraoperative genetic assay system,^5^ a *BRAF* p. V600E mutation was identified (Figure 1F). Considering the potential for targeted therapy with dabrafenib (BRAF inhibitor) and trametinib (MEK inhibitor), as well as the need to preserve hippocampal memory function, we elected to perform a partial resection sparing the hippocampal-involved portion (Figure 1G;  Figure S1C). Postoperatively, the patient experienced no neurological complications, and follow-up neuropsychological testing demonstrated preserved memory function, including verbal memory. At 16 months postoperatively, volumetric analysis revealed slow progression of the contrast-enhancing lesion; however, the growth pattern was clearly distinct from that of a rapidly enlarging tumor. Consequently, the patient was initiated on molecular targeted therapy with dabrafenib and trametinib.

## Pathology and Molecular Diagnosis

Histopathological analysis revealed a low-grade oligodendroglioma-like tumor with strong CD34 immunopositivity and no evidence of calcification. Histological features indicative of pleomorphic xanthoastrocytoma (PXA), astroblastoma, or other neuroglial tumors was absent. In addition, no aggressive pathological features, such as necrosis and microvascular proliferation, were observed (Figure 1H). In contrast, the Ki-67 labeling index was 20%, indicating a relatively high proliferative potential. Immunohistochemistry showed positive reactivity for Oligo2, S100, Nestin, ATRX, and BRAF^V600E^, and negative reactivity for IDH1^R132H^ and p53 (Figure 1H; Figure S1D).

Molecular genetic analysis demonstrated the absence of *IDH1/2* mutations and 1p/19q co-deletion, while identifying *BRAF* p. V600E and *TERT* c.-124C>T (C228T) mutations (Figure 1I; Figure S1E-F). Functional evaluation in our translational research platform,^6^ demonstrated that dabrafenib, but not trametinib, significantly suppressed cell viability in patient-derived primary tumor cultures at day 3 (Figure 1J).

**Figure 2.**
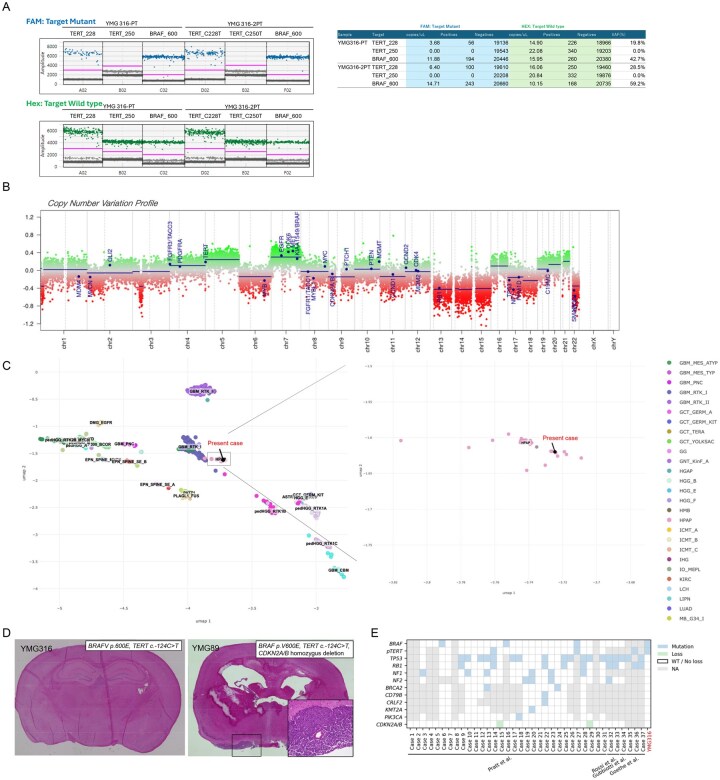
Integrated Molecular Characterization of the Tumor. (A) Detection of *TERT* promoter and *BRAF* p. V600E mutations using droplet digital PCR (ddPCR): FAM fluorescence plots indicate the presence of mutant alleles (blue dots), while HEX fluorescence plots show wild-type alleles (green dots) for TERT_228, TERT_250, and BRAF_600 in samples YMG316-PT and YMG316-2PT. Quantitative analysis shows the presence of the *BRAF* p. V600E mutation in both samples, with variant allele frequencies (VAFs) of 42.7% and 59.2%, respectively. *TERT* c.-124C>T mutation was detected with lower VAFs (19.8% and 28.5%), while *TERT* c.-146C>T was not detected (VAF 0.0%). These results confirm co-occurrence of *BRAF* p. V600E and *TERT* c.-124C>T mutations in the tumor specimens. (B) Genome-wide copy number variation profile: Chromosomal gains (green) and losses (red) are plotted across chromosomes 1 to 22 and X/Y. Significant copy number alterations are observed, with blue dots highlighting 29 brain tumor–relevant gene loci. (C) UMAP plots of DNA methylation profiles: The left panel shows an UMAP plot based on DNA methylation profiles of reference CNS tumor samples. The present case is indicated by a black dot and is located within the HPAP cluster. The right panel focuses on the region containing HPAP samples, showing that the present case clusters within the HPAP group, supporting its classification as HPAP., supporting its classification as HPAP. Color coding is based on NCI-Bethesda CNS tumor classifier (Laboratory of Pathology, NCI), accessed via Methylscape (https://methylscape.ccr.cancer.gov/, accessed October 5th, 2025). (D) Hematoxylin and eosin staining of non-xenograft formed mouse brain (YMG316, left) and xenograft formed mouse brain (YMG89, right). Inset, high magnification. (E) Oncoplot showing genomic profiling of 38 HPAP tumors.

Further analysis using a Comprehensive Genomic Profiling (CGP, GeneMineTOP) identified non-pathogenic alterations in *KEL* (p.P669H, VAF 10.8%) and *STIM1* (p.E434*, VAF 13.7%), in addition to pathogenic mutations in *BRAF* (p.V600E, VAF 56.9%) and *TERT* (c.-124C>T, VAF 31.8%). The tumor mutation burden was low, calculated at 1.6 mutations per megabase. To validate the apparent disparity in VAFs between *BRAF* and *TERT* mutations, droplet digital PCR was performed for distinct tumor specimens (Figure 2A). This confirmed *BRAF* p. V600E VAF of approximately 40–60% and a *pTERT* VAF of 20–30%, consistent with the CGP results. Based on the integrated histopathological and molecular features, we initially considered this tumor to represent a rapidly progressive form of PLNTY; however, its clinical characteristics were atypical for this tumor type.

DNA methylation profiling using the Heidelberg Brain Tumor Classifier (v12.8) did not yield a confident match to any established WHO-defined tumor class (Figure S2A). Copy number profiling demonstrated multiple chromosomal alterations, including loss of chromosomes 13–15, 17–18, and 22 (Figure 2B), without evidence of *CDKN2A/B* homozygous deletion. The *MGMT* promoter methylation was predicted with high confidence (estimated score: 0.94, Figure S2B). Notably, classification using the NCI-Bethesda DNA methylation classifier (v2) yielded a high-confidence match HPAP, with a calibrated score of 0.969 (Figure S2C). The uniform manifold approximation and projection (UMAP) confirmed the present case clusters within the HPAP group (Figure 2C). To validate these findings, we performed t-distributed stochastic neighbor embedding (t-SNE) analysis incorporating 2801 reference samples^7^ and 23 HPAP cases [GSE195567]. The present case was clustered within the HPAP reference group (Figure S2D). Finally, to evaluate tumorigenic potential, we attempted to establish orthotopic patient-derived xenograft models. Xenograft formation was assessed using the human-specific STEM121 antibody. As a control, we used YMG89, a highly aggressive *BRAF* p. V600E–mutant high-grade glioma cell line harboring *pTERT* mutation and *CDKN2A/B* homozygous deletion.^8^ In contrast to YMG89, which reliably formed xenografts, no xenograft formation was observed in mice implanted with YMG316 cells, in line with distinct clinical characteristics (Figure 2D;  Figure S2E).

## Discussion

In the 2021 World Health Organization (WHO) Classification of Tumors of the Central Nervous System (WHO CNS 5), a group of circumscribed astrocytic gliomas was formally defined. This category includes pilocytic astrocytoma, high-grade glioma with piloid features, PXA, subependymal giant cell astrocytoma, chordoid glioma, and astroblastoma, *MN1*-altered.^9^ Following this framework, HPAP has been recognized as a novel methylation-defined class of relatively circumscribed gliomas.^1^

Pratt et al. first proposed the descriptive yet representative term “HPAP” for a distinct DNA methylation class encompassing 31 cases identified from approximately 14,000 CNS tumor samples through unsupervised clustering via the NCI-Bethesda classifier. This methylation class is closely related to, but distinct from PXA and PLNTY.^1^ Histologically, HPAP demonstrates heterogenous features, overlapping with both low- and high-grade gliomas, including PLNTY, astroblastoma, glioblastoma, anaplastic ependymoma, and anaplastic PXA, as well as glioneuronal tumors.^1-4^ Among these, PLNTY is a low-grade epilepsy-associated tumor that predominantly affects young adults and usually follows an indolent clinical course. PLNTY is pathologically characterized by oligoastrocytoma-like morphology with diffuse strong CD34 immunoreactivity. Importantly, rapid progression of PLNTY is rarely observed.^10^

In the present case, t-SNE analysis using the Heidelberg Brain Tumor Classifier incorporating with 23 HPAP cases revealed that the HPAP cluster, including the present case, was located near PXA cases, while phenotypic features of the tumor aligned more closely with PLNTY. These features included the presence of a *BRAF* p. V600E mutation, oligodendroglioma-like cellular morphology, strong CD34 immunopositivity, and the absence of *IDH1/2* mutation and 1p/19q co-deletions. However, the tumor exhibited atypical biological behavior not characteristic of classic PLNTY: a high Ki-67 labeling index, absence of calcification, emergence of contrast enhancement, and rapid progression on serial imaging. These findings contrast with the typically indolent nature of PLNTY.^3^ Moreover, the absence of epilepsy throughout the clinical course, unusual for PLNTY, further suggests a distinct biological background. Importantly, copy number analysis derived from DNA methylation profiling demonstrated chromosome 13 loss, a recurrent alteration in HPAP,^1^ thereby supporting classification of this case as HPAP. Taken together, the histological, molecular, and clinical features indicate that this tumor represents HPAP rather than conventional PLNTY.

To further characterize the genetic features of HPAP, we reviewed 38 cases, including the present case (Figure 2E;  Tables S1). The most frequent mutation was *TP53* (18/29, 62.1%), followed by *RB1* (9/29, 31.0%) and *NF1* (6/24, 25.0%). The *BRAF* p. V600E mutation was detected in 4/31 (13.0%) cases. Among the 38 HPAP cases, PLNTY-like morphology was observed in 3 cases (7.9%), which was less common than glioblastoma-like (8/38, 21.1%) or astroblastoma-like features (6/38, 15.8%). Notably, PLNTY-like morphology occurred in two *NF1*-mutant cases and one *BRAF* p. V600E-mutant case. Since PLNTY is typically characterized by either *BRAF* p. V600E mutation or *FGFR2/3* fusion,^11^^,^^12^ these findings suggest that *BRAF* p. V600E mutation may underlie PLNTY-like morphology in HPAP, a possibility that warrants further investigation. Of note, primary cultured cell exhibited sensitivity to BRAF inhibition, underscoring the potential utility of molecular targeted therapy in *BRAF*-mutant HPAP.

Importantly, the present tumor harbored a *pTERT* mutation with a lower VAF than *BRAF* p. V600E, raising the possibility of a *pTERT*-mutant subpopulation within the *BRAF*-mutant tumor; however, such interpretations must be made cautiously given the limitations of clinical next generation sequencing-based VAF estimates. Clinically, the tumor demonstrated a prolonged indolent phase followed by rapid growth outside the hippocampus. The co-occurrence of *BRAF* p. V600E and *pTERT* mutations may synergistically contribute to tumor aggressiveness through MAPK-pathway activation and telomerase regulation, consistent with findings from large-scale studies of adult *BRAF*-mutant gliomas.^13^ Previous reports have identified *pTERT* mutations in three HPAP cases, including one recurrent tumor.^1^^,^^2^ Together with the present case, these findings suggest that *pTERT* mutation is not rare in HPAP. In addition to *pTERT* mutation, the presence of chromosomal instability may further accelerate tumor progression in HPAP. Further studies are needed to clarify how combinations of genomic alterations contribute to malignant transformation in this rare tumor.

In contrast to *BRAF* p. V600E–mutant high-grade gliomas such as epithelioid glioblastoma, which frequently harbor *pTERT* mutation in combination with *CDKN2A/B* homozygous deletion,^8^ the prognosis in *BRAF*-mutant HPAP may be relatively more favorable. This is consistent with the tumor’s demonstrated ability to form xenografts.^8^ Interestingly, after resection of the progressed *pTERT*-mutant tumor, the residual hippocampal lesion remained relatively stable for 16 months, further supporting the presence of intra-tumoral genomic heterogeneity. However, because histopathological examination of the initial MRI-detected lesion was not performed, it cannot be concluded that this tumor arose through malignant transformation of a pre-existing low-grade lesion.

In summary, we report a unique case of *BRAF*-mutant HPAP with a *pTERT* mutation, which demonstrated progressive disease over long-term follow-up. Although the histopathological and genomic features overlapped with those of PLNTY, the clinical course was distinctly more aggressive.

## Supplementary Material

vdag008_Supplementary_Data

## Data Availability

The data underlying this article are available from the corresponding author upon reasonable request.
