# Supplementary material for: BRAF-mutant high-grade glioma with pleomorphic and pseudopapillary features (HPAP): A PLNTY mimic demonstrating tumor progression during longitudinal follow-up
Source: Neurooncol Adv. 2026 Jan 18;8(1):vdag008. doi: 10.1093/noajnl/vdag008 (PMC12962799; doi:10.1093/noajnl/vdag008)
Supplement: vdag008_Supplementary_Data [file vdag008_supplementary_data.zip › HPAP_Supplementary Figure R1.pdf]

Supplementary Figure 1

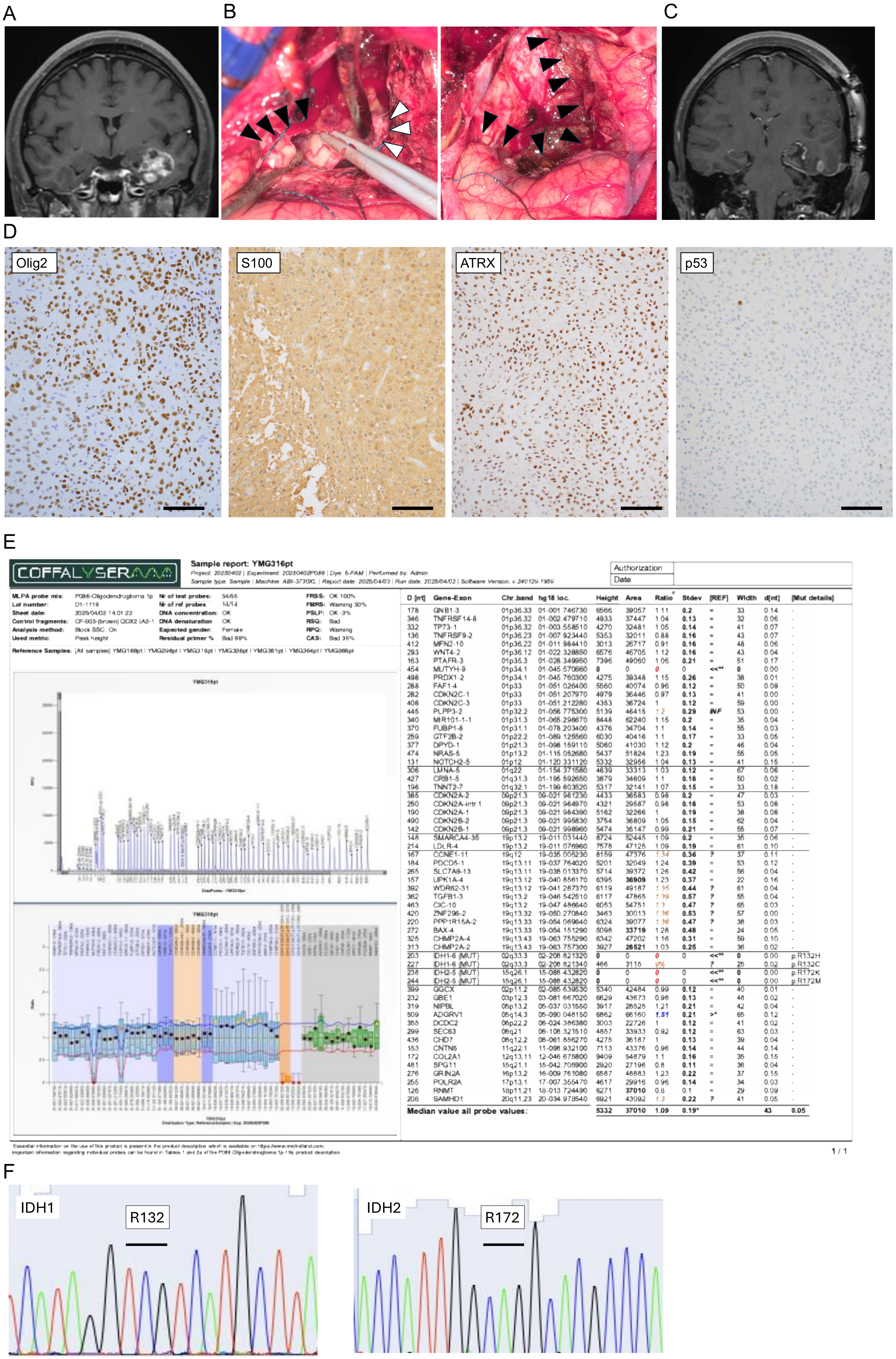

Supplementary Figure 1: (A) Preoperative coronal T1-weighted contrast-enhanced MRI.**(B)** Intraoperative image: Black arrowheads indicate the parahippocampal gyrus, and white arrowheads denote the hippocampus. **(C)** Postoperative coronal T1-weighted contrast-enhanced MRI. **(D)** Immunohistochemistry for the indicated proteins. Bars, 100μm. **(E)** MLPA analysis of sample YMG316pt performed using Coffalyser. The upper panel displays the electropherogram of probe signals, and the lower panel shows normalized copy number ratios. Ratios near 1.0 indicate normal copy number, while values below 0.7 suggest deletions. In the lower panel, probes corresponding to chromosome arms 1p (e.g., 1p36, 1p32) and 19q (e.g., 19q13) show ratios close to 1.0, without evidence of combined whole-arm loss. These findings are further supported by the numerical table on the right, where the ratio values for 1p and 19q probes remain within the normal range. The results do not support the presence of 1p/19q codeletion and are not consistent with oligodendroglioma. **(F)** Sanger sequencing of IDH1 and IDH2 hotspot regions. The chromatograms show wild-type sequences at codon R132 of IDH1 (left) and codon R172 of IDH2 (right), indicating the absence of pathogenic mutations in either gene.

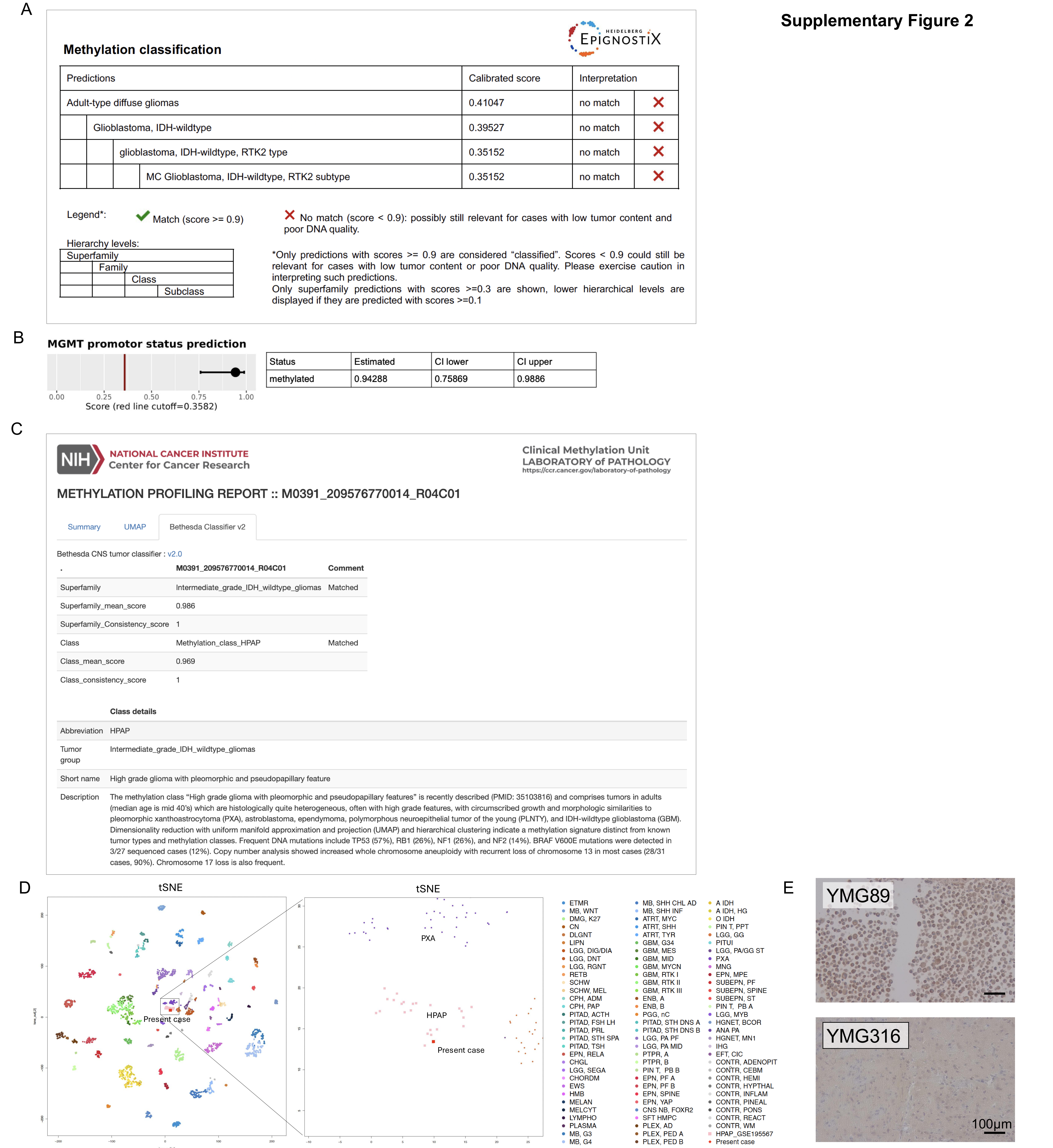

A

Methylation classification

Predictions				Calibrated score	Interpretation	
Adult-type diffuse gliomas				0.41047	no match	✗
	Glioblastoma, IDH-wildtype			0.39527	no match	✗
		glioblastoma, IDH-wildtype, RTK2 type		0.35152	no match	✗
			MC Glioblastoma, IDH-wildtype, RTK2 subtype	0.35152	no match	✗

Legend*: ✓ Match (score >= 0.9) ✗ No match (score < 0.9): possibly still relevant for cases with low tumor content and poor DNA quality.

Hierarchy levels:

Superfamily			
	Family		
		Class	
			Subclass

*Only predictions with scores >= 0.9 are considered "classified". Scores < 0.9 could still be relevant for cases with low tumor content or poor DNA quality. Please exercise caution in interpreting such predictions.
Only superfamily predictions with scores >=0.3 are shown, lower hierarchical levels are displayed if they are predicted with scores >=0.1

B

MGMT promotor status prediction

Status	Estimated	CI lower	CI upper
methylated	0.94288	0.75869	0.9886

C

NATIONAL CANCER INSTITUTE Center for Cancer Research

Clinical Methylation Unit LABORATORY of PATHOLOGY https://ccr.cancer.gov/laboratory-of-pathology

METHYLATION PROFILING REPORT :: M0391_209576770014_R04C01

Summary UMAP Bethesda Classifier v2

Bethesda CNS tumor classifier : v2.0

.	M0391_209576770014_R04C01	Comment
Superfamily	Intermediate_grade_IDH_wildtype_gliomas	Matched
Superfamily_mean_score	0.986	
Superfamily_Consistency_score	1	
Class	Methylation_class_HPAP	Matched
Class_mean_score	0.969	
Class_consistency_score	1	

	Class details
Abbreviation	HPAP
Tumor group	Intermediate_grade_IDH_wildtype_gliomas
Short name	High grade glioma with pleomorphic and pseudopapillary feature
Description	The methylation class "High grade glioma with pleomorphic and pseudopapillary features" is recently described (PMID: 35103816) and comprises tumors in adults (median age is mid 40's) which are histologically quite heterogeneous, often with high grade features, with circumscribed growth and morphologic similarities to pleomorphic xanthoastrocytoma (PXA), astroblastoma, ependymoma, polymorphous neuroepithelial tumor of the young (PLNTY), and IDH-wildtype glioblastoma (GBM). Dimensionality reduction with uniform manifold approximation and projection (UMAP) and hierarchical clustering indicate a methylation signature distinct from known tumor types and methylation classes. Frequent DNA mutations include TP53 (57%), RB1 (26%), NF1 (26%), and NF2 (14%). BRAF V600E mutations were detected in 3/27 sequenced cases (12%). Copy number analysis showed increased whole chromosome aneuploidy with recurrent loss of chromosome 13 in most cases (28/31 cases, 90%). Chromosome 17 loss is also frequent.

Supplementary Figure 2: (**A**)Methylation classification results using the Heidelberg Classifier: According to the Heidelberg CNS tumor classifier (v12.8), the present case did not match any known methylation classes with high confidence (all calibrated scores < 0.9). Although the tumor showed some similarity to adult-type diffuse gliomas, particularly the glioblastoma, IDH-wildtype (RTK2 subtype), none of the predicted classes exceeded the classification threshold. **(B)** *MGMT* promoter methylation status estimation: The calculated score was 0.94288, which is well above the established threshold (0.3582, red line), indicating a methylated MGMT promoter. **(C)** Methylation Classifier Report Identifying HPAP (Bethesda CNS classifier v2.0): Methylation profiling using the Bethesda CNS classifier v2.0 identified the present tumor as belonging to the methylation class "HPAP" (High-grade glioma with pleomorphic and pseudopapillary features), with high confidence (class mean score = 0.969). **(D)** t-distributed stochastic neighbor embedding (t-SNE) visualization of genome-wide DNA methylation profiling. (Left) Unsupervised clustering based on DNA methylation data from the 2801 reference cohort and 23 HPAP samples. HPAP cluster is indicated pink squares labeled "HPAP_GSE195567" and the present case is highlighted in red. (Right) Enlarged view showing that the present case clusters within the HPAP group, supporting its classification as HPAP. (**E**) STEM121 Immunohistochemistry for YMG316 (upper) and YMG89 (lower) implanted mouse brain.
